# The Important Role of Immunotherapies in Acute Myeloid Leukemia

**DOI:** 10.3390/jcm8122054

**Published:** 2019-11-22

**Authors:** Jochen Greiner

**Affiliations:** 1 Department of Internal Medicine, Diakonie Hospital, 70176 Stuttgart, Germany; greiner@diak-stuttgart.de; 2 Department of Internal Medicine III, University Hospital of Ulm, 89081 Ulm, Germany

This series on immunotherapies in acute myeloid leukemia (AML) aims to give readers new insights on established but also emerging immunotherapeutic approaches for AML patients. The therapeutic landscape in AML is rapidly changing, and several drugs have been developed and approved such as first and second generation FLT3 inhibitors [1,2,3], IDH1 and 2-inhibitors [4,5], demethylating agents, liposomal cytarabine and daunorubicin (CPX-351) [6], venetoclax [7,8] and the hedgehog pathway inhibitor glasdegib. However, relapse after intensive chemotherapy or allogeneic hematopoietic stem cell transplantation is one of the major obstacles impeding the complete elimination of all AML cells [9]. Thus, although the median overall survival for AML patients has increased, it still remains relatively low [10]. 

Therefore, immunotherapeutic approaches might be an option to prevent disease relapse and to eliminate leukemic cells or leukemic stem cells (LSC) that survive intensive treatment approaches. The efficacy of immunotherapeutic approaches has become ever more evident in solid tumors, especially immune-checkpoint inhibitors that are routinely used in several solid tumor entities, but also lymphoma [11,12]. 

Our focus in this special issue is different strategies of immunotherapeutic approaches in AML.

Some of the immunotherapies in the treatment of AML, such as allogeneic hematopoietic stem cell transplantation (HSCT) and donor lymphocyte infusion (DLI), have been part of routine clinical practice in the treatment of AML for a long time, whereas other immunotherapeutic approaches have only recently entered clinical practice or need to be further developed. A key aspect is the mechanisms underlying the cure of AML patients, which are based on the graft-versus-leukemia (GvL) effect, in which allogeneic T cells recognize target antigens on malignant cells by T cell approaches including DLI. An effective and well-tolerated regimen for HSCT in patients with AML and MDS is the FLAMSA-RIC regimen, and therefore novel data of this approach are presented in this issue [13].

It is very appropriate to utilize DLI after allogeneic HSCT to prevent relapse, to prolong progression-free survival, to establish full donor chimerism, and to restore the GvL effect in patients with hematological malignancies. There are different strategies to use DLI in a therapeutic setting for the treatment of morphological relapse, and also for prophylactic use in AML/MDS and DLI administered preemptively. There is also the approach of antigen-directed immunogenic and specifically stimulated and modified DLI as well as virus-specific donor T cells and third-party DLI [14].

DC-based immunotherapies also have the potential to bring about demonstrable clinical responses in AML patients, although there has not been a complete breakthrough for this type of therapy until today. Van Acker et al. have highlighted different DC strategies in AML [15].

Leukemia-associated antigens (LAAs) represent immunogenic structures to target LSC [16,17], and LAA might be relevant for the elimination of malignant cells by cytotoxic T lymphocytes. Therefore, LAAs might be a good target for specific immunotherapeutic approaches. Several LAAs have been identified in the context of malignant hematological diseases [16,18,19], and in clinical phase I/II peptide vaccination trials, some LAAs showed immunological as well as clinical responses [20,21,22,23]. 

In this special issue, we also elucidate antibody-based therapies in AML, such as T cell activating antibodies including immune-checkpoint inhibitors and diverse monoclonal antibodies [11,12,24]. Immune-checkpoint inhibitors have changed clinical treatment algorithms of malignant diseases such as malignant melanoma, lung cancer, as well as lymphoma. Today, immune-checkpoint inhibitors are not yet established in the routine treatment of AML but should be considered as further immunotherapeutic options in the future, especially in the context of allogeneic stem cell transplantation [24]. Further antibody-directed approaches such as unconjugated, toxin-conjugated, radio-conjugated, and multivalent formats of antibody-based therapy, are demonstrating the potential of a diverse leukemia-derived antibody strategy which is already established in acute lymphoblastic leukemia and are summarized in one section of this issue [25].

Chimeric antigen receptor T cells (CARs) are highly effective in the treatment of refractory and relapsed acute lymphoblastic leukemia, to some lower extent in aggressive lymphoma, but also in multiple myeloma [26]. However, early CAR-T cell approaches are also being tested in AML with interesting target structures, and these strategies are described in this issue [27]. Immune responses are complex and are also influenced by T cell cross-talk and communication by cytokines and the communication of leukemic cells with their microenvironment, as presented by Reikvam et al. [28] in this issue. 

All of these aspects emphasize the high potential of immunotherapeutic approaches to improve the survival of AML patients in the future, where combination therapies utilizing immunotherapeutic drugs could represent further innovation strategies to further improve the treatment of AML.
